# Medical College Advertisements

**Published:** 1882-09-20

**Authors:** 


					﻿Medical College Advertisements.—Read the advertise-
ments in this issue of the Southern Medical College, Atlanta, and
the Jefferson Medical College, Philadelphia, representative Insti-
tutions of the two sections of the Union.
				

